# Precision Teaching to Develop Key Word Sign Skills in Practitioners in Intellectual Disability Services: A Proof‐of‐Concept Study

**DOI:** 10.1111/jar.70188

**Published:** 2026-01-25

**Authors:** Athanasios Vostanis, Oliver Douglas Payne, Anthony Cox, Jill Bradshaw

**Affiliations:** ^1^ Tizard Centre University of Kent Kent UK; ^2^ Ambitious About Autism The Pears National Centre for Autism Education London UK; ^3^ Intellectual Disabilities Research Institute University of Birmingham Birmingham UK

**Keywords:** AAC, augmentative and alternative communication, fluency, standard celeration chart

## Abstract

**Background:**

People with intellectual disabilities frequently communicate through Key Word Signs (KWS). Although various methods have trained practitioners to sign, they have not led to increased use. This study evaluated whether Precision Teaching would lead to improved outcomes.

**Method:**

Practitioners were divided into experimental (*n* = 4) and control conditions (*n* = 5) using a multiple baseline across participants design. Experimental participants received Precision Teaching for 60 signs three times a week for 14 weeks. Control participants had completed a one‐day training event via Makaton at least 1 year before. Naturalistic observations were conducted for all participants to measure signing while supporting service users.

**Results:**

Experimental participants demonstrated fluency, emergent application, and maintenance of improvements. They also signed more than control participants when supporting clients.

**Conclusion:**

Precision Teaching can lead to increased usage of KWS under naturalistic conditions. However, training in isolation might be inadequate. Additional elements, such as practice leadership, are required.

## Lay Summary

- Many people with intellectual disabilities rely on Key Word Signs (KWS), but standard staff training methods often fail to increase their use.
- This study used Precision Teaching to train four staff members in 60 KWS.
- Staff showed fluent signing, maintained their skills, and signed more than colleagues who had received standard training when supporting service users.
- Precision Teaching may improve training outcomes, but additional support, like practice leadership, is needed to enhance sign use in practice.

## 1 Introduction

People with intellectual disabilities are likely to experience difficulties with both receptive and expressive communication (Smith et al. 2020). Augmentative and Alternative Communication (AAC) approaches are one method of supporting communication for people with a range of complex communication needs (Light and McNaughton 2012), and the introduction of these approaches can lead to improved communication between the person with intellectual disabilities and their communication partners (Light and Mcnaughton 2015). While a range of ‘aided’ and ‘unaided’ AAC approaches exist, Key Word Signing, an ‘unaided’ option, where the sentence is spoken while simultaneously signing the key words in the sentence, is commonly used in services for people with intellectual disabilities (Meuris et al. 2014; Rombouts et al. 2017). Key Word Signing can support individuals in developing spoken words (Launonen 2019), supports comprehension as the sign focuses attention on the most important words in the sentence (Rombouts et al. 2017), and often reduces message complexity. Message complexity is often reduced as the communication partner simplifies their verbal communication to include the Key Word Signs (KWS) they know. The thinking required to do this often also means that the production of the message is somewhat slower (Loncke et al. 2012).

Staff use of AAC in services for people with intellectual disabilities is often limited (Beadle‐Brown et al. 2016; Iacono et al. 2019). For example, Beadle‐Brown et al. (2016) found that over 80% of adults with severe intellectual disabilities in social care settings received only verbal communication, despite staff having recognised that 60% of people needed some additional support in terms of AAC. Crowe et al. (2022) highlight the importance of communication partners in interventions as they can help integrate the intervention within daily contexts.

### 1.1 What Factors Might Influence the Use of AAC?

When AAC is used, achieving a successful interaction will depend on the knowledge, skills, and attitudes of both individuals involved in the exchange (Kent‐Walsh and Mcnaughton 2005). When people live in social care settings, this includes the knowledge and skills of both the person with an intellectual disability and the staff member. Hanley et al. (2023) discussed that the implementation of AAC systems was impacted by knowledge, attitudes to the approach, commitment to working within a partnership, and a suitable method of AAC. In addition, Moorcroft et al. (2019) found that support provided by professionals and personal factors (including socio‐economic status and culture) were influential.

Research has shown that whilst some staff might be skilled communicators, others require support and training. Kent‐Walsh and Mcnaughton (2005) identified eight stages of communication partner instruction, including: pretest and commitment (assessment of current use of AAC and commitment to learning the approach); description of the strategy; demonstration of the strategy; verbal practice; controlled practice and feedback; advanced practice and feedback; post‐test and commitment; generalisation.

Training in KWS in the United Kingdom typically takes place through packaged approaches such as Signalong or Makaton, which offer structured training broken into different levels of complexity (i.e., Phases or Levels) and accompanying manuals and resources (see https://signalong.org.uk/ and https://makaton.org/). Exploring training in KWS more broadly, it is typically delivered as one‐day workshops (Smidt et al. 2019) and may include video feedback (Rombouts et al. 2016) and be face‐to‐face or online (Smidt et al. 2025). During training, KWS instructors typically demonstrate how each sign should be performed and allow staff members to imitate the sign, providing confirmatory or corrective feedback (Rombouts et al. 2017). Although all approaches lead to improvements in KWS knowledge and performance, maintaining these effects is challenging, and further research is needed. Notably, evidence suggests that those attending a one‐day workshop are likely to lose some of the signs they learnt post‐training (e.g., 6 to 12 weeks later; Le Van et al. 2019; Smidt et al. 2019).

### 1.2 Precision Teaching

One system that could prove particularly useful for developing KWS skills is Precision Teaching. This system supports practitioners in designing, implementing, and monitoring the effects of the training provided, allowing them to engage in dynamic and strategic decision‐making (Evans et al. 2021; Reagan 2025; Vostanis 2025). That way, training outcomes are optimised, making the most of the available time to deliver the training. Precision Teaching has a rich history of helping students improve their academic skills, such as English and Mathematics (Brosnan et al. 2018; McTiernan et al. 2016, 2018; Newsome et al. 2024). However, it has been primarily implemented by Precision Teachers, rather than professionals from other disciplines (Diffley et al. 2025; McTiernan et al. 2022). Similarly, it has been directly implemented with individuals with intellectual disabilities and has not been widely used to train the professionals or caregivers supporting those individuals (Ramey et al. 2016). Despite that limitation, Precision Teaching has been applied in other areas due to its versatility, including professional skills (Pampino et al. 2005), training medical students (Lydon et al. 2019), and training professionals who support individuals who have behaviour support plans (Branch et al. 2018).

**FIGURE 1:**
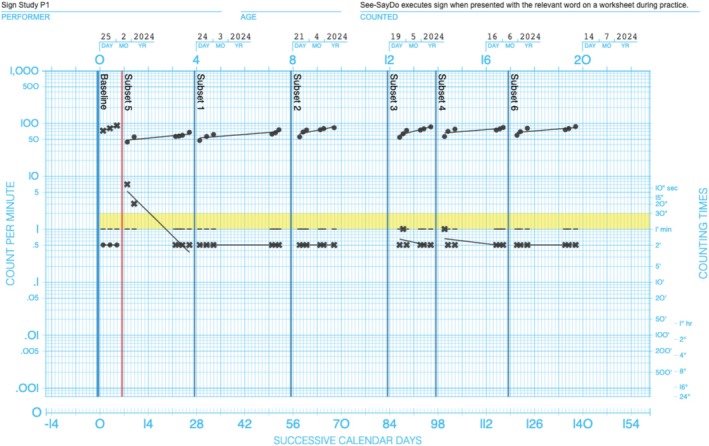
An example of the daily‐per‐minute standard celeration chart. This chart presents data from Participant 1 during practice of the first skill. The closed circles depict correct responses, and the crosses depict incorrect responses. The yellow band shows the maximum number of incorrect responses that would be deemed acceptable during training (i.e., no more than 2). The small black bars on the chart, called record floors, indicate the duration of the timing, which is shown on the right y‐axis (i.e., 1 min). Data points below the record floors indicate zero (see Kubina Jr and Yurich 2012, for more information, as well as www.celeration.org).

Precision Teaching (PT) follows a framework of (a) pinpointing the skills to be targeted for training, (b) delivering the training, (c) collecting and charting the data, and (d) evaluating progress and acting accordingly (see Table 1; Evans et al. 2021). One of the hallmarks of PT is the use of a family of standardised visual displays that have been ‘built‐for‐purpose’ called the Standard Celeration Charts (see Figure 1). They enable Precision Teachers to evaluate both performance and learning (Calkin 2005). Notably, performance and learning are considered separate terms in PT and are not used interchangeably. Performance is defined as one's ability to engage in a skill at a given point in time and is quantified through the frequency of correct and incorrect responses. Learning is defined as a change in one's performance across time and is quantified by a measure called celeration. Celeration provides information on the direction and magnitude of performance changes across time. Increasing performances are labelled as accelerating, and their magnitude is presented as ratios using the multiplication symbol. For example, if someone doubled their correct responses, from Monday to Friday, their acceleration would be depicted as ×2.00 (100% increase; which is what Precision Teachers consider the golden standard; Johnson and Street 2012). Similarly, decreasing performances are labelled as decelerating, and their magnitude is presented using the division symbol. For example, if someone's incorrect responses decreased by half across a week, they would be decelerating at a rate of ÷2.00 (50% reduction; which is what Precision Teachers strive for; Johnson et al. 2021; Kubina Jr and Yurich 2012).

**TABLE 1:** The primary steps of precision teaching when implemented with key word signing.

Pinpointing the Skills Targeted for Training	PTers identify the skills that should be targeted for practice and create pinpoints that help them precisely measure their participants' performance with these skills. Pinpoints are created by combining learning channel sets, movement cycles, and adding additional context. Learning Channel sets, such as See‐Say, Hear‐Do, See‐Write, help specify the modality of instruction. For example, See‐Say indicates that the participant will *See* a question and *Say* the answer. Movement cycles, such as *Says Word*, *Writes Letter*, *Executes Sign*, help identify the smallest unit of measurement. For example, one word or one sign to be recorded as correct or incorrect. A pinpoint also includes additional context, such as “using worksheets during reading practice.” A complete pinpoint includes all three elements. For example, for KWS training we pinpointed the skill as: See‐Say. Do executes sign when randomly presented on a worksheet during practice. The pinpointed skills are typically scored as correct or incorrect and the focus of PTers is to increase the correct responses and decrease the incorrect ones. (see Kubina Jr and Yurich 2012, for a detailed analysis of how to create pinpoints).
Delivering the Training	During training PTers employ various strategies depending on the skill they are focusing on. For example, different activities are used when practising reading versus mathematical skills. Typically, PTers combine untimed activities that focus on the accuracy of responding (acquisition stage of learning) and timed activities that focus on developing the natural pace of responding (fluency stage of learning). For example, PTers might deliver untimed practice with KWS where they model each sign and offer confirmatory or corrective feedback on the accuracy of each sign's execution before transitioning to a short timing of 1 min where they allow participants to practice building their natural pace of responding by letting them respond freely without interrupting them while collecting data on their performance and offering feedback at the end of the timing. This approach aligns instruction to the stages of learning (see Jimenez et al. 2021, for a detailed explanation of the stages of learning).
Collecting and Charting the Data	During practice sessions, PTers expect participants to collect data on their correct and incorrect responses. Participants can either collect their data on datasheets or plot their data immediately on the SCC. This way they receive continuous feedback about their performance. For example, during KWS training, participants can write down the number of correct and incorrect signs after each 1‐min timing they complete. They can then plot their best score of the day on the SCC allowing them to receive feedback about their daily performance. (see Johnson et al. 2021, for a detailed account of the process).
Evaluating Progress and Acting Accordingly	As data collection is ongoing, PTers make moment‐to‐moment decisions about their participants' progress. This can be within‐session decision making, such as adjusting the session to provide more untimed practice if performance does not seem to be improving across timings or fading the untimed practice and offering more timings to help their participants build fluency, if accuracy seems to be in place, but responding remains slow and hesitant. PTers also engage in between‐sessions analysis, where they evaluate the data across days and decide whether the instruction can continue as is, requires additional amendments, or can be concluded due to the participants meeting the mastery criteria. For example, during KWS training, PTers might notice that incorrect signs remain high despite increases in correct responding, which would essentially show that the participant is performing signs faster but are not necessarily becoming more accurate in their responding. In that case, they might adjust the session to offer more untimed practice and provide more opportunities for modelling the sign and providing corrective feedback before focusing on developing their participant's fluency. Ultimately, it is this recursive process of monitoring the effects of one's instruction or training that makes their teaching (or training) more precise, which is why the field is called Precision Teaching. (see Kubina Jr and Yurich 2012, for a detailed account of how to engage in within‐ and between‐sessions analysis).

Abbreviations: KWS, Key Word Signing; PTers, Precision Teachers; SCC, Standard Celeration Chart.

These two primary measures enable Precision Teachers to collect information on two primary training outcomes: fluency and agility (Binder 1996; Meyer et al. 2021). Fluency is defined as accurate performance that has a natural, effortless pace (Binder 1996). Developing fluency is considered essential for achieving true mastery in skills and has been linked with a series of ‘by‐products’ which are typically assessed by Precision Teachers, namely (a) endurance, defined as one's ability to perform consistently for extended practice periods, (b) stability, defined as one's ability to perform consistently in the face of distractions, (c) maintenance, defined as one's ability to perform consistently after a period of no practice, and (d) application defined as one's ability to apply their skills to perform more complex ones and under varying contexts (Fabrizio and Moors 2003; Kostewicz et al. 2020).

Agility is one's ability to ‘learn how to learn faster’. Although it has not attracted the same attention as fluency in the PT literature, it is an essential training outcome that Precision Teachers strive for (Meyer et al. 2021). Agility enables participants to master related content more efficiently. For example, if someone is practising multiplication tables, they would demonstrate agility if they mastered the more advanced tables more efficiently than the earlier ones (Meyer et al. 2021). Considering that practice is typically provided across multiple sets of related content, such as math facts, music notes, or KWS, designing instruction that results in learners being more agile is considered vital in PT as it would allow practitioners to optimise their training outcomes further. Such a consideration would be crucial for training delivered to practitioners in services for individuals with intellectual disabilities, where there is limited time available for additional training.

Despite its varied applications, PT has not been employed to develop KWS skills for practitioners in intellectual and developmental disabilities services. Therefore, this study wanted to evaluate whether providing training within a PT framework would lead to beneficial outcomes for practitioners working in services for people with intellectual disabilities. Specifically, this study addressed these research questions:

1. What is the effect of combining PT with fluency‐based training on participants' KWS skills?
2. Will participants achieve fluency in those skills by the end of the training?
3. How will the training affect participants' use of signs in their daily work routines?

## 2 Method

### 2.1 Research Framework

**FIGURE 2:**
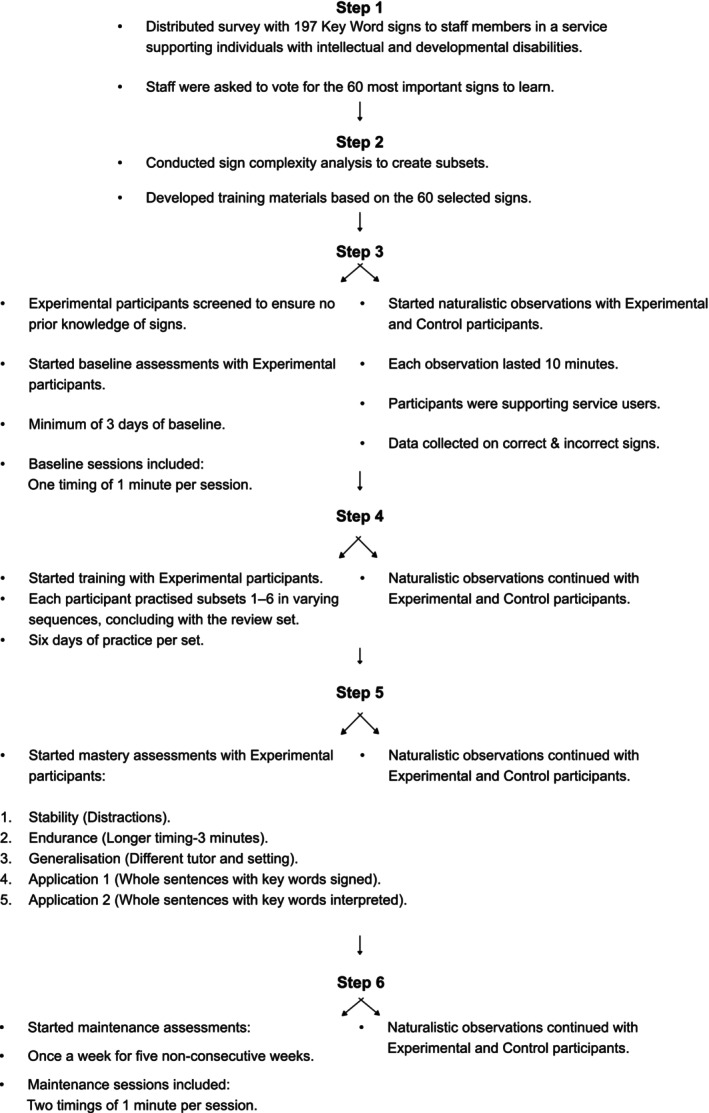
The study's primary steps.

This study employed a single‐case methodology. Single‐case designs employ the baseline logic and continuous performance monitoring, making them well‐suited for identifying functional relations with small sample sizes. Various designs exist, such as the reversal, alternating treatments design, and multiple baseline design, and they are all widely used in the behavioural literature (Kazdin 2016; Ledford et al. 2022). Four experimental participants underwent multicomponent training aimed at fluency outcomes for 60 KWS, three times a week, completing a total of 42 training sessions over 14 weeks. Each session averaged 10 min, and data were collected on correct and incorrect responses. Additionally, five control participants were recruited, all of whom had completed Makaton level 1 training at least 1 year before the study began. Naturalistic observations were carried out throughout the study for all participants as they supported their clients, with an average of 21 observations per participant, each lasting 10 min. Correct and incorrect uses of KWS were recorded (see Figure 2).

### 2.2 Participants

Ethical approval was obtained from the University of Kent ethics committee, and this applied to both the participants who completed the study and the practitioners who were surveyed regarding the signs relevant to the service. Purposive sampling was used to identify possible services. Operated by the same organisation at a single site in the south of England, the services comprised three residential houses and a day service focused on supporting adults with intellectual disabilities. Once the service agreed to participate, information about the study was sent to support workers, inviting them to take part.

#### 2.2.1 Inclusion Criteria

To be allocated to the PT/experimental conditions, staff needed to:

1. Be 18 years or older.
2. Have no previous official training in KWS.
3. Have prior knowledge of no more than 5 out of the 60 signs targeted for training in this project.
4. Be able to commit to receiving training at their workplace 3 days a week (for no more than 30 min per session) for 5–6 months.

Control participants needed to:

1. Be at least 18 years old.
2. Have previously undergone formal or informal training in KWS.

Of the 10 practitioners who agreed to participate (see Table 2), one experimental participant (P5) withdrew because their working hours were incompatible with the study. In total, we recruited four participants for the experimental conditions and five as controls.

**TABLE 2:** Participants' demographic information.

Participant	Age	Gender	Ethnicity	Years of work experience	Prior key word sign training
P1	30s	Male	Black	2–5	No
P2	40s	Male	Black	2–5	No
P3	20s	Female	Other‐Turkish	2–5	No
P4	30s	Female	White	1–2	No
C1	40s	Female	White	5+	Makaton Level 1
C2	30s	Female	Black	1–2	Makaton Level 1
C3	20s	Female	Other‐Turkish	2–5	Makaton Level 1
C4	20s	Female	White	2–5	Makaton Level 1
C5	20s	Female	White	1–2	Makaton Level 1

*Note:* P1–P4 were the participants who received Precision Teaching. C1–C5 were the control participants. P5 withdrew from the study within the first few weeks, so their data was not reported. All participants provided direct support to service users. Some of the information is provided in aggregate form (e.g., work experience) to prevent participants from being identified.

### 2.3 Key Word Signs

Staff in the organisation completed a survey to identify the most relevant signs for the service users from 197 key words within Makaton Levels 1 and 2. Fifteen practitioners responded, and the top 60 signs were selected. The 60 signs were divided into six subsets of ten to make practice more manageable. To help participants discriminate the different signs effectively, each set contained signs that were different in terms of movement, placement, hand shape, and the number of hands used (see Table 3). Although the subsets included the same signs, the order of practice was randomised. Thus, participants practised the same 60 signs but in a different sequence (see Table 4). Each subset was practised three times a week for 2 weeks. Once all subsets were trained, a review set was developed that included all 60 signs. In this way, participants practised all signs, including similar ones. Participants practised with this review set three times a week for 2 weeks.

**TABLE 3:** Key word signs included in each subset.

Subset 1	Subset 2	Subset 3	Subset 4	Subset 5	Subset 6
Hot (feeling)	To give	To swim	To wash	Mother/Mummy	Clean
Apple	I	Please	Sister	Father/Daddy	What
Outside	Good	To go	To sleep	To come	Car
Coffee	Chocolate	To eat	Mine	Yes	You
Chicken	Home	To drink	Thank you	Tea (a)	Where
Cake	Sorry	More	Dinner	Drink (a)	Water (drink)
Bed	To give	Shower (a)	Television	Door	My
Brother	Cold	Me	Cup	No	Goodbye
Cereal	Hello/Hi	Spoon	To sit	Dirty	Friend
To walk	Juice	Please	To shower	Man	Food

*Note:* These Key Word signs were chosen out of 197 in total based on professionals' responses to the survey distributed before the study commenced. The review set included all 60 Key Word signs. The Key Word signs were presented in random order on the worksheets to avoid rote responding.

**TABLE 4:** The sequence with which subsets were presented to participants during the training.

P1	P2	P3	P4
Subset 5	Subset 4	Subset 4	Subset 3
Subset 1	Subset 2	Subset 1	Subset 2
Subset 2	Subset 5	Subset 5	Subset 6
Subset 3	Subset 1	Subset 3	Subset 4
Subset 4	Subset 3	Subset 6	Subset 1
Subset 6	Subset 6	Subset 2	Subset 5
Review Set	Review Set	Review Set	Review Set

*Note:* Each subset included the same stimuli for all participants. The only difference was in the sequencing. Each subset was trained three times a week for 2 weeks. The review set consisted of all 60 signs and was also trained for 2 weeks.

### 2.4 Materials

#### 2.4.1 Training Materials for Untimed Practice

A video camera, placed in the training room, recorded each training session to assess inter‐observer agreement (IOA) and procedural fidelity. For each subset, a Google Slides file was created with 10 slides showing GIFs of the instructor performing the target signs in random order. Pressing the space bar revealed the sign's name, allowing participants to learn unfamiliar ones. The model signed silently to prevent lip‐reading; however, participants were encouraged to vocalise the signs during practice. The demonstrator had 5 years' experience and Makaton Levels 1 and 2 training. In the review set, six slide decks, each containing ten of the 60 signs, were used across six training days over 2 weeks, ensuring practice with all signs.

#### 2.4.2 Training Materials for Timed Practice

Separate worksheets were created for each participant and each subset, including the review set along with duplicate follow‐along sheets the instructor used to score their performance. Each worksheet was generated using MS Excel, which facilitated word randomisation and helped guard against serial learning. Participants were provided with more words than they could possibly respond to during each timing, preventing artificial performance ceilings. Avoiding such ceilings is considered essential in PT, where the focus is on one's natural pace of responding that indicates fluency (Binder 1996). Participants were instructed to read the word and execute the relevant sign whilst saying the word.

#### 2.4.3 Materials for the Assessment of Mastery

For endurance and stability, we used the same worksheets as the ones used for the review set practice. To evaluate generalisation, we developed separate worksheets for each participant consisting of 150 words per page, arranged in random order.

To assess application, two different resources were developed. First, a series of sentences were generated using the 60 targeted signs. Second, GIFs were created similar to those used for untimed practice, where the instructor signed without vocalising whole sentences instead of isolated key words.

#### 2.4.4 Participant Datasheets and Charts

A separate datasheet was created for each subset to record participants' performance during timed practice. Participants were given their results after each timing. Standard Celeration Charts were also used during practice to provide participants with visual feedback about their performance over time. Participants were encouraged, by the instructor, to record their best score of the day at the end of each practice session on a daily per‐minute chart, enabling them to evaluate their progress over time.

#### 2.4.5 Naturalistic Observation Datasheets

Data collection sheets were created on paper for the naturalistic observations. These sheets included the 60 signs being trained. The signs were scored by placing a ‘C’ when participants signed correctly and an ‘X’ when they signed incorrectly.

### 2.5 Experimental Design

The study employed a concurrent multiple baseline across participants design (Carr 2005), with participants randomly assigned to one of five tiers.

### 2.6 Dependent Variable

This study measured two dependent variables (DVs). The first variable assessed participants' performance during structured practice sessions, while the second assessed their performance during naturalistic observations. The DVs were operationally defined using pinpointing, a core component of PT's framework that integrates movement cycles and instructional (or learning) channels.

DV1: See‐Say. Do executes sign when randomly presented on a worksheet during practice.

DV2: Free^1^‐Say. Do execute signs while communicating with a service user.

During practice, the frequency of correct and incorrect responses per minute was recorded. In naturalistic observations, correct and incorrect responses were documented throughout the observation and then converted into a count per minute. A correct response was scored if the participants executed the sign with complete accuracy. If the sign was not executed with complete accuracy, the response was scored as incorrect.

Finally, for the two application assessments, the number of correct and incorrect sentences was measured instead of isolated words. A correct sentence was scored if all signs were executed with complete accuracy.

### 2.7 Procedure

#### 2.7.1 Baseline

**FIGURE 3:**
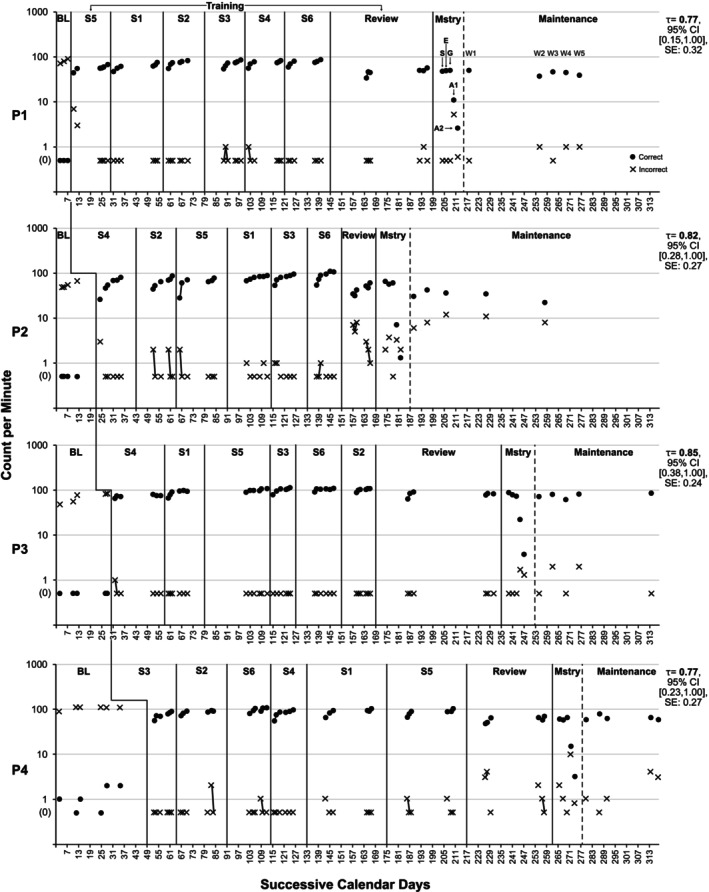
Precision teaching participants' performance executing key word signs during training. The sequence of subsets was randomly assigned to each participant. Upon completing all subsets, participants underwent training covering all 60 signs (i.e., Review stage). The by‐products of fluency were assessed during the mastery assessment. Each datum point represents a separate fluency assessment: S = Stability; E = Endurance; G = Generalisation; A1 = Application (participants executed sentences; Also see Figure 7); and A2 = Application (participants interpreted 15 GIFs where full sentences were signed; Also See Figure 7). Although the mastery assessment was conducted on the same day, the data were plotted separately to enable visual analysis. For A1 and A2, the number of correct and incorrect sentences is represented rather than the number of isolated signs measured in the other assessments. The five maintenance assessments were conducted over separate weeks, depending on participants' availability. Successive calendar days were used to illustrate breaks in training due to annual leave or participant absences. TAU‐BC was calculated by comparing baseline and maintenance conditions using the following website: https://jepusto.shinyapps.io/SCD‐effect‐sizes/. The y‐axis is a ratio scale without a true zero value, and zeros were plotted according to the conventions of standard celeration charting (Kubina Jr and Yurich 2012). BL = Baseline; S1‐6 = Subset 1–6; Mstry = Mastery; W1‐5 = Weeks 1–5.

**FIGURE 4:**
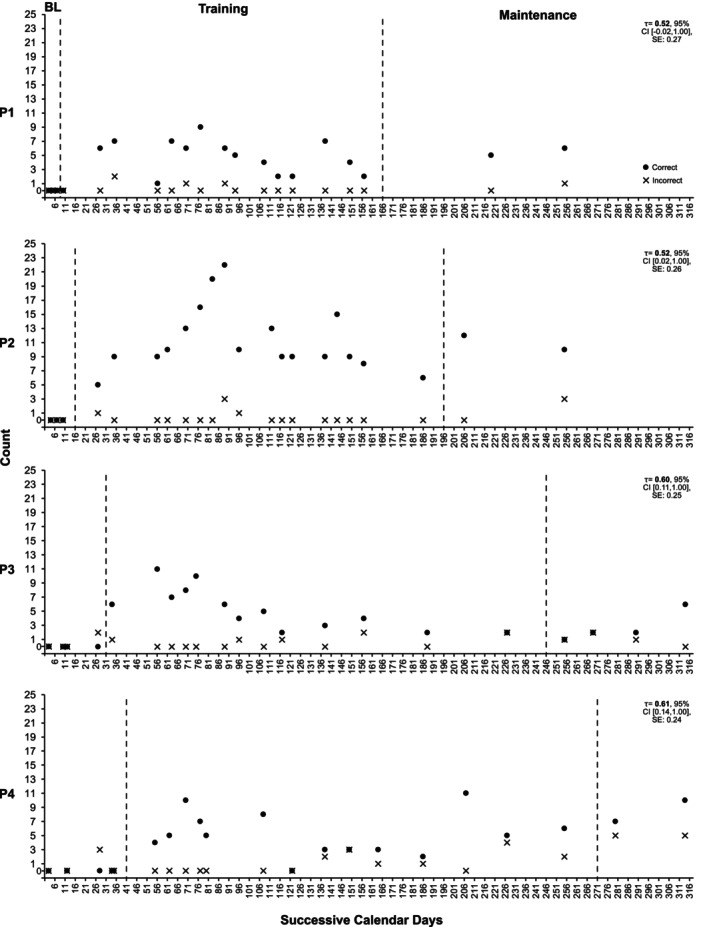
Precision teaching participants' execution of key word signs while supporting service users. These data were collected during 10‐min naturalistic routine observations within the service, with the days and times of observation being randomly determined. Dashed condition change lines have been added to indicate the introduction of the training and maintenance conditions, respectively. TAU‐BC was calculated by comparing baseline data with the combined training and maintenance conditions using the following website: https://jepusto.shinyapps.io/SCD‐effect‐sizes/. BL, Baseline.

Each participant's ability to perform and vocalise signs was evaluated across multiple sessions. Each participant was assigned to a Tier within the design, with Participant 1 introduced to the practice first (i.e., Tier 1), and subsequent participants introduced gradually in accordance with the design's conventions. A Tier denoted the sequence in which participants received the instruction and was displayed as a separate panel in the primary figures (see Figures 3 and 4). All participants completed at least 3 days of baseline assessments, which increased for participants in Tiers 2–4 according to the conventions of the experimental design employed. The first baseline session occurred on the same day for all participants, while subsequent sessions varied in terms of times and days. There were no instances of multiple baseline assessments occurring within the same day.

Although participants were selected based on having no prior sign training, sign knowledge was assessed. Participants were informed that they were not expected to know the signs. Participants were asked to perform and vocalise the relevant signs. No feedback was given about their performance. Once the timing was complete, participants were thanked, and the session concluded. Baseline sessions lasted, on average, 2:17 min (range: 01:29–03:26) for all experimental participants.

#### 2.7.2 Training: Precision Teaching and Fluency‐Based Practice

The training provided was multicomponent and included video modelling, untimed practice, timed practice, and graphing. Participants practised three times a week with an instructor. Each session began with a round of untimed practice using the previously described Google Slides. Participants sat in front of a laptop and started the slideshow. On each slide, they were expected to see the model, perform, and vocalise the sign. If participants executed and vocalised the sign correctly, they pressed the space bar twice to move to the next slide. If participants did not know the sign, they were prompted to click the space bar once to reveal the sign's name. Then, the instructor engaged in a corrective procedure called Model‐Lead‐Test. They first modelled and vocalised the sign, then asked participants to repeat it with them, and finally requested that participants execute and vocalise it independently.

When participants completed a round of untimed practice, they conducted four successive 1‐min timings. During each timing, participants were provided with their datasheet and relevant worksheet. They were asked to read each word on the worksheet and execute and vocalise each sign. Participants were asked to be as accurate as possible and perform at their natural pace. They were allowed to respond for the whole duration of the timing without any interruptions. At the end of the timing, they were informed of their score, and the instructor provided additional feedback on the incorrect signs using the Model‐Lead‐Test procedure. Participants were then guided to transfer their data to their datasheets. This process was repeated until participants completed all four successive timings. At the end of the practice, participants were asked to transfer the day's best score on a digital Daily Per Minute Standard Celeration Chart provided on an iPad using the AimStar Pro application (Xcelerate Innovations 2021). Training sessions lasted, on average, 10:01 min (range: 07:27–17:51) for all experimental participants.

#### 2.7.3 Absence Protocol

When participants missed two sessions out of the three in a week due to sickness or personal reasons, the week was reset, and they started from scratch the week after. P1 had a week reset during subset 6, P2 had a week reset during subset 1, P3 had a week reset during subset 5, and P4 had no week reset.

#### 2.7.4 Mastery Assessment

At the end of the training, the by‐products of fluency described in PT were evaluated, namely stability, endurance, generalisation, application, and maintenance. For stability, participants performed a 1‐min timing while a colleague next to them, with prior KWS training, also practised signs, executing and vocalising them. During endurance, participants were asked to engage in a 3‐min timing. For generalisation, participants completed a 1‐min timing with the colleague mentioned above, in a different location, using the generalisation worksheet. Application was assessed in two ways. First, participants engaged in a 3‐min timing where they were expected to sign whole sentences using the worksheet designed for this task. Second, participants were given short GIFs of the instructor signing sentences and were asked to say what each sentence conveyed. In this case, we timed how long it took them to interpret 15 sentences presented as GIFs on a laptop. Finally, we also assessed maintenance. Specifically, we allowed for 1 week without practice, then evaluated the participants’ ability once a week for five non‐consecutive weeks, depending on their availability. During maintenance assessments, participants engaged in two 1‐min timings, just as they did during the baseline. This way, they had the opportunity to participate in a ‘warm‐up’ timing to adjust for the period without practice. Maintenance assessments lasted, on average, 02:45 min (range: 02:27–02:56) for all experimental participants.

#### 2.7.5 Naturalistic Observations

From the start of the study, naturalistic observations were carried out as part of the service's routine monitoring for both experimental and control participants. These observations occurred once a week for each participant on randomly selected weeks, days, and times. Each observation lasted 10 min and was conducted when the participants supported their clients in the service. For both experimental and control participants, an average of 21 observations (range: 20–22) were conducted throughout the study.

### 2.8 Inter‐Observer Agreement and Procedural Fidelity

All teaching sessions were recorded on video. Data were gathered for an equal number of sessions for both IOA and procedural fidelity. During the baseline phase, the number of evaluated sessions varied among participants according to the experimental design. Specifically, 33% of baseline sessions were scored for P1, 25% for P2, 40% for P3, and 33% for P4. For all participants, 33% of the teaching sessions were evaluated for each subset (Subsets 1–6) and 33% of the teaching sessions in the review set. Additionally, we scored the mastery assessment and 40% of the maintenance sessions conducted for each participant.

The third author, a certified behaviour analyst with 7 years of experience, who had received KWS training at their workplace, scored the videos for IOA. The average agreement was 100% (range: 96%–100%) across all participants and conditions. To ensure procedural fidelity, four checklists were developed to align with the study's primary conditions. Average procedural fidelity was 100% (range: 98%–100%).

Finally, we also calculated IOA for the naturalistic observations using point‐to‐point agreement. In this case, two staff members of the organisation collected data as part of the routine observations conducted within the service. For the experimental participants, IOA was collected for an average of 41.72% (range: 38.10%–45%) of the observations conducted. Average agreement was 92% (range: 86%–100%). For the control participants, IOA was collected for an average of 31.14% (range: 30%–33.33%) of the observations conducted. Average agreement was 92% (range: 86%–98%).

### 2.9 Social Validity

At the end of the study, participants were invited to an interview to discuss their experiences with the training and its impact. Three out of four participants agreed to be interviewed.

### 2.10 Data Analysis

A series of metrics were calculated using PrecisionX (Central Reach 2019), a software similar to AimStar Pro (Xcelerate Innovations 2021) that provides digital Standard Celeration Charts and additional PT metrics. We calculated the level that shows average performance in each condition, using the geometric mean to minimise the influence of outliers (Everitt and Howell 2005). We calculated celeration, which is typically expressed as a ratio: acceleration (×) and deceleration (÷). Daily celeration was calculated for baseline and training phases, while weekly celeration was used for maintenance assessments, employing the least‐squares regression method. Celeration values were converted into percentages for ease of interpretation. We calculated bounce, a measure of variability in performance over time, expressed as a ratio (×). A bounce value up to ×3.00 indicates stable, consistent performance. We also calculated the Level Change Multiplier, a ratio quantifying changes in average performance across conditions (e.g., baseline vs. training), calculated by dividing the highest by the lowest value. Multiplication or division symbols indicate increases or decreases. Finally, the baseline‐corrected Tau (Tau‐BC) was calculated using an online tool (Pustejovsky et al. 2024; Tarlow 2017). Tau‐BC is a nonparametric effect size that accounts for monotonic trends in baseline data and corrects them if necessary (Tarlow 2017). Depending on the values produced, the effects can be considered small (0–0.65), medium (0.66–0.92), or large (0.93–1.00).

## 3 Results

### 3.1 Precision Teaching Practice

Participant 1's signing ability increased by a factor of ×86.28 from baseline to maintenance, meaning they were 86 times more proficient by the end of the study. Participant 2 improved by ×64.14, Participant 3 by ×150.58, and Participant 4 by ×64.41. The training produced moderate effect sizes, and all participants' performance during maintenance remained substantially higher than in baseline (see Figure 3).

**FIGURE 5:**
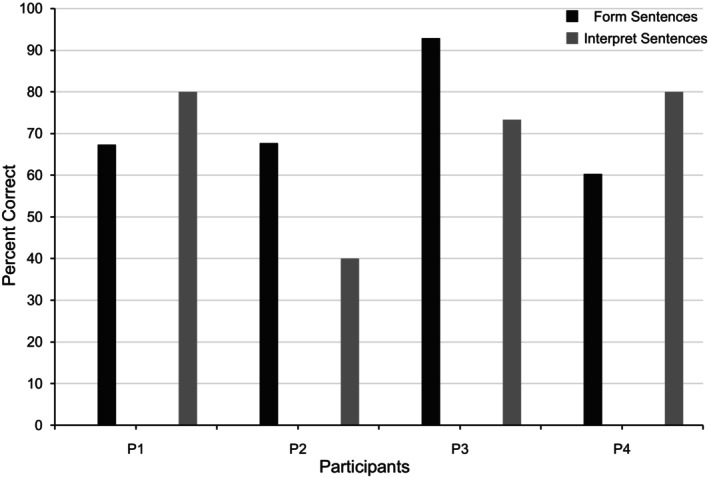
Participants' accuracy in the two application checks conducted during the mastery assessment. These assessments were conducted after the completion of training during the mastery assessment phase. For the first application check (i.e., form sentences; labelled as A1 in Figure 2), participants were required to sign and vocalise complete sentences rather than isolated signs. They engaged in a 3‐min timing and signed as many sentences as possible. For the second application check (i.e., interpret sentences; labelled as A2 in Figure 2), participants watched 15 GIFs where the instructor signed whole sentences without vocalisation, minimising the risk of inadvertent prompting. Participants reviewed the GIFs at their own pace and attempted to interpret each sentence. The total time taken to complete all 15 GIFs was recorded. Participants had no prior practice with either skill and did not receive feedback during the assessments.

Participant 1 exhibited no correct signs during baseline (see Figure 3, first panel). During training, they averaged 65.05 correct signs per minute (range: 34–86) with 0.31 incorrect signs (range: 0–7) across all subsets and the review stage. Their celeration of correct signs was low, averaging ×1.14 (range: ×1.06–×1.32), and average bounce was ×1.29 (range: ×1.20–×1.40; see Table 5). During the mastery assessment, they maintained their performance, indicating fluency. During application, they were 67.35% accurate with forming sentences and 80% with interpreting sentences, suggesting emergent but not full application (see Figure 5). During maintenance, they averaged 43.40 correct signs per minute (range: 37–50) and 0.6 incorrect signs (range: 0–1). Correct signs decelerated by ÷1.10, with a ×1.30 bounce.

**TABLE 5:** Precision teaching participants' performance metrics during key word sign training.

Participants	Metrics	Subsets							
		5	1	2	3	4	6	R	M
1	CC	×1.11 (+11%)	×1.09 (+9%)	×1.24 (+24%)	×1.32 (+32%)	×1.09 (+9%)	×1.09 (+9%)	×1.06 (+6%)	÷1.10 (−9%)
	CI	÷2.81 (−64%)	×1.00 (0%)	×1.00 (0%)	÷1.26 (−21%)	÷1.14 (−12%)	×1.00 (0%)	×1.06 (+6%)	×1.36 (+36%)
	BC	×1.20	×1.30	×1.30	×1.20	×1.30	×1.30	×1.40	×1.30
	BI	×1.90	×1.00	×1.00	×1.90	×1.90	×1.00	×1.90	×2.00

Participants	Metrics	Subsets							
		4	2	5	1	3	6	R	M
2	CC	×1.91 (+91%)	×1.46 (+46%)	×1.21 (+21%)	×1.17 (+17%)	×1.38 (+38%)	×1.57 (+57%)	×1.50 (+50%)	÷1.24 (−19%)
	CI	÷2.35 (−57%)	÷1.33 (−25%)	÷1.29 (−22%)	÷1.11 (−10%)	÷1.66 (−40%)	÷1.16 (−14%)	÷3.68 (−73%)	×1.11 (+11%)
	BC	×1.50	×1.20	×2.00	×1.10	×1.30	×1.40	×1.30	×1.60
	BI	×3.30	×4.20	×2.90	×2.30	×1.70	×2.00	×2.20	×1.90

Participants	Metrics	Subsets							
		4	1	5	3	6	2	R	M
3	CC	×1.03 (+3%)	×1.23 (+23%)	×1.11 (+11%)	×1.24 (+24%)	×1.07 (+7%)	×1.16 (+16%)	×1.01 (+1%)	×1.11 (+11%)
	CI	÷1.09 (−8%)	×1.00 (0%)	×1.00 (0%)	×1.00 (0%)	×1.00 (0%)	×1.00 (0%)	×1.00 (0%)	÷1.33 (−25%)
	BC	×1.20	×1.30	×1.10	×1.20	×1.10	×1.10	×1.40	×1.40
	BI	×1.90	×1.00	×1.00	×1.00	×1.00	×1.00	×1.00	×4.40

Participants	Metrics	Subsets							
		3	2	6	4	1	5	R	M
4	CC	×1.32 (+32%)	×1.06 (+6%)	×1.19 (+19%)	×1.34 (+34%)	×1.07 (+7%)	×1.06 (+6%)	×1.05 (+5%)	÷1.06 (−6%)
	CI	×1.00 (0%)	×1.26 (+26%)	×1.13 (+13%)	×1.00 (0%)	÷1.09 (−8%)	÷1.02 (−2%)	÷1.19 (−16%)	×3.44 (+244%)
	BC	×1.20	×1.20	×1.30	×1.40	×1.40	×1.30	×1.30	×1.40
	BI	×1.00	×3.10	×2.00	×1.00	×1.90	×2.40	×4.90	×2.50

*Note:* Subsets are presented in the order they were practised for each participant. Celeration metrics were converted into percentages for clarity. A multiplication symbol (×) denotes an acceleration in performance over time, whereas a division symbol (÷) indicates deceleration. An acceleration of ×2.00 is regarded as the gold standard in Precision Teaching, while ×1.30 represents the minimum expected weekly growth (Kubina Jr and Yurich 2012). Celeration values can be interpreted as the rate of learning participants demonstrated during training. Bounce values (i.e., variability) were not converted into percentages. Performance is considered stable if bounce does not exceed ×3.00. All metrics are based on daily calculations, except for maintenance, which was calculated across weeks.

Abbreviations: BC, Bounce of Corrects; BI, Bounce of Incorrects; CC, Celeration of Corrects; CI, Celeration of Incorrects.

Participant 2 exhibited no correct signs during baseline (see Figure 3, second panel). During training, they averaged 67.24 correct signs (range: 26–108) with 0.95 incorrect signs (range: 0–8). Their celeration of correct signs averaged ×1.46 (range: ×1.17–×1.91), with a bounce value of ×1.20 (range: ×1.10–×2.00). During the mastery assessment, they demonstrated emergent fluency and emergent application with 67.74% accuracy in forming and 40% in interpreting sentences. During maintenance, they averaged 32.80 correct signs (range: 22–42) and 9 incorrect signs (range: 6–12). Correct signs decelerated by ÷1.24, with a ×1.60 bounce.

Participant 3 exhibited no correct signs during baseline (see Figure 3, third panel). During training, they averaged 91.81 correct signs (range: 63–112) with 0.02 incorrect signs (range: 0–1). Their celeration averaged ×1.12 (range: ×1.01–×1.24) with a ×1.20 (range: ×1.10–×1.40) bounce. During the mastery assessment, they demonstrated fluency and emergent application with 92.86% accuracy in forming and 73.33% in interpreting sentences. During maintenance, they averaged 75.80 correct signs (range: 61–85) and 0.80 incorrect signs (range: 0–2). Correct signs accelerated by ×1.11, with a ×1.40 bounce.

Participant 4 exhibited an average of 1 correct sign per minute (range: 0–2) during baseline (see Figure 3, fourth panel). During training, they averaged 81.79 correct signs (range: 48–108) with 0.38 incorrect signs (range: 0–4). Their celeration averaged ×1.16 (range: ×1.05–×1.34) with a ×1.30 bounce (range: ×1.20–×1.40). During the mastery assessment, they demonstrated fluency and emergent application with 60.27% accuracy in forming and 80% in interpreting sentences. During maintenance, they averaged 64.80 correct signs (range: 59–79) and 1.80 incorrect signs (range: 0–4). Correct signs decelerated by ÷1.06 with a ×1.40 bounce.

### 3.2 Naturalistic Observations

While in baseline conditions, experimental participants did not use any KWS while supporting service users. Following the introduction of training, all participants demonstrated an increase in the average number of correctly used signs. Participant 1 increased their average correct signs by a factor of ×7.40, as calculated using the level change multiplier (see Figure 4, first panel). This equates to a 640% increase compared to baseline, meaning they used signs approximately seven times more often. Specifically, their usage increased from an average of 0 correct signs per 10 min during baseline to an average of 5 per 10 min (range: 0–9) across training and maintenance conditions. Participant 2 improved by ×21.00, from 0 to 11 (range: 5–22; see second panel). Participant 3 increased by ×7.60, from 0 to 5 (range: 1–11; see third panel). Finally, Participant 4 increased by ×9.00, from 0 to 6 (range: 0–11; see fourth panel).

**FIGURE 6:**
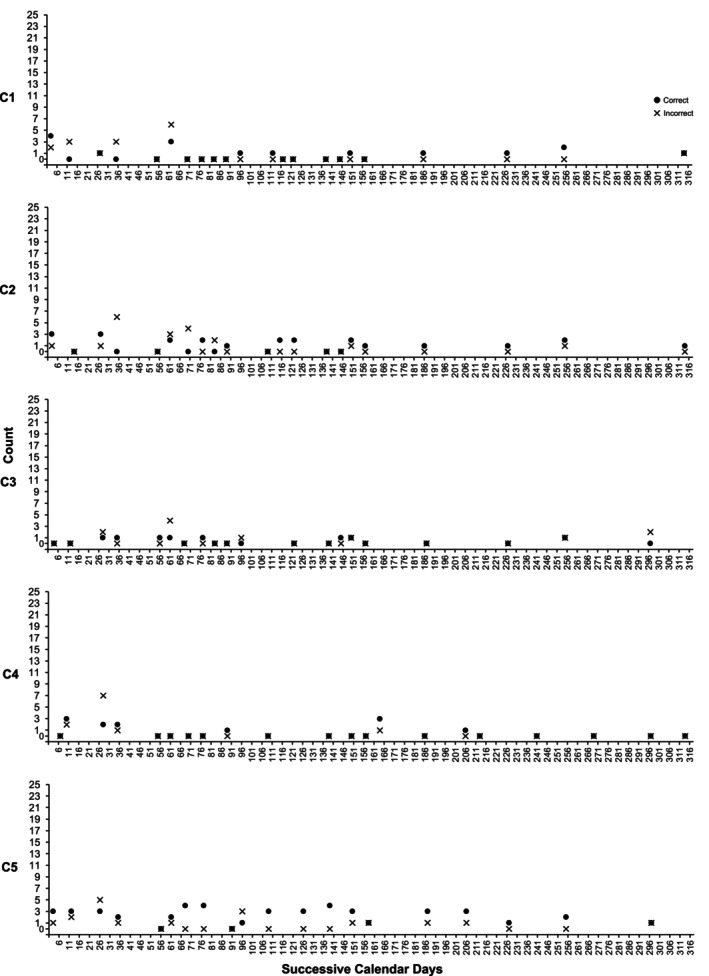
Control participants' execution of key word signs while supporting service users. These data were collected during 10‐min naturalistic routine observations within the service, with the days and times of observation being randomly determined. These participants had previously completed formal training in using Key Word signs to support their clients and served as the control participants in this study.

Despite being trained in KWS, control participants demonstrated minimal usage while supporting service users. Control 1 averaged 1 correct sign per 10 min (range: 0–4; see Figure 6, first panel), Control 2 averaged 1 correct sign (range: 0–3; see second panel), Control 3 averaged 0 correct signs (range: 0–1; see third panel), Control 4 averaged 1 correct sign (range: 0–3; see fourth panel), and Control 5 averaged 2 correct signs (range: 0–4; see fifth panel).

**FIGURE 7:**
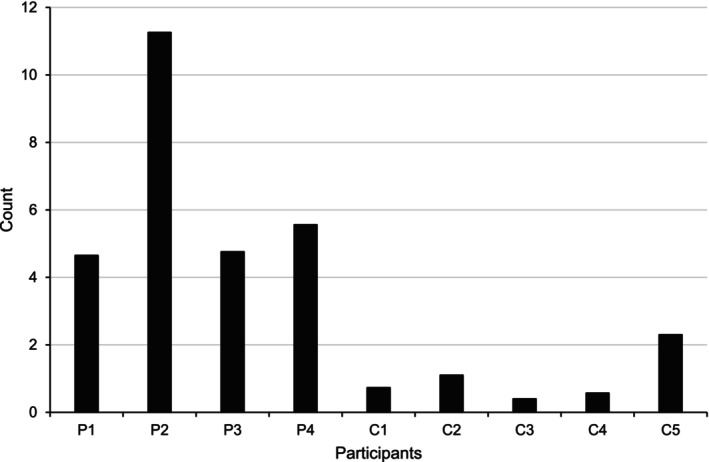
Comparison of the average correct usage of key word signs between precision teaching and control participants when supporting service users. This graph presents the average number of correct signs recorded during naturalistic observations conducted in the service. Participants P1–P4 received Precision Teaching, while C1–C5 were control participants who had previously been trained in Key Word signs through commercially available mainstream training packages. For Precision Teaching participants, averages were calculated from the point they began Precision Teaching training, as they had no prior knowledge of the signs. Consequently, naturalistic observations conducted during baseline were excluded from these calculations.

Overall, naturalistic observations indicated that experimental participants used signs more frequently than control participants when supporting service users (see Figure 7).

### 3.3 Social Validity

Participants interviewed reported feeling satisfied with the quality of the training, which they said increased their knowledge and confidence in supporting service users' communication needs through KWS. A detailed analysis of findings will be presented in a separate publication.

## 4 Discussion

This study aimed to answer a series of research questions, namely (a) What is the effect of combining PT with fluency‐based training on participants' KWS skills? (b) Will participants achieve fluency in those skills by the end of the training? (c) How will the training affect participants' use of signs in their daily work routines?

Regarding the first research question, this study evaluated the effect of combining PT and fluency‐based training on participants' KWS skills. Overall, the results are promising and in line with the existing PT literature (Gist and Bulla 2022; McTiernan et al. 2022; Ramey et al. 2016). Participants increased their ability from having no prior knowledge of the signs to an average performance exceeding 60 signs per minute during instruction. Overall, the instruction seems to have led to considerable positive improvements for all four participants. Therefore, this study adds to the existing PT literature and demonstrates a novel application of PT. Although PT has been used to develop language skills before (Cihon 2007; Cihon et al. 2017; Thakore et al. 2021), to our knowledge, this is the first study to focus on KWS and staff rather than service users.

Regarding the second research question, it seems that three out of four participants achieved fluency in the skill (i.e., accuracy plus a natural, effortless pace), with only P2 demonstrating the need for more training. Achieving fluency in skills is important as it will make it more likely that participants will recruit them when supporting service users. For example, the fact that three out of four participants seemed to be able to perform in the face of distractions (i.e., stability), for longer periods of time (i.e., endurance), and across different individuals and settings (i.e., generalisation), suggests that they will be able to recruit these skills when supporting the service users. Considering how busy natural settings are, it is crucial to demonstrate those by‐products of fluency if staff members are to use signs throughout the day. Although the by‐products of fluency are not widely assessed outside the PT literature, other studies have demonstrated their emergence (Vostanis et al. 2023, 2022, 2024). Therefore, this study adds to that literature and draws further attention to the importance of developing not only accurate but also fluent skills. Various calls have been made in the PT literature about the importance of focusing on fluency, but unfortunately, accuracy‐based measures, typically in the form of percent correct, tend to be the norm (Evans et al. 2021; Fabrizio and Moors 2003). This fact is problematic as accuracy only addresses the first stage of learning (acquisition). In other words, while accuracy in executing signs is important, it is equally important to execute them at a natural pace that links to the second stage of learning, which is fluency (Binder 1996; Jimenez et al. 2021). Therefore, for a comprehensive delivery of instruction, practitioners should have opportunities to address both primary stages of learning to achieve true mastery (Bulla et al. 2024).

Another noteworthy finding is that participants demonstrated emergent application. Application refers to recombining skills one has mastered to perform a more complex one (Kostewicz et al. 2020). In this case, we evaluated application by examining whether, without explicit training, participants would (a) form whole sentences using KWS and (b) interpret whole sentences when signed by someone else. Although participants did not demonstrate full application, they did show an emergence of the skills as demonstrated by their accuracy in forming and interpreting sentences. This fact suggests that training isolated KWS prepares participants to engage in more complex signing, but that explicit training might be required to develop the skill fully. This result aligns with similar findings in the literature where training basic skills led to spill‐over improvements to more complex ones (Kostewicz et al. 2020; Vostanis et al. 2024). Therefore, it would be worth evaluating how much explicit training would be required for participants to develop their ability to sign and interpret whole sentences. Such a skill could prove particularly useful when supporting individuals with intellectual disabilities who communicate via KWS, as they are meant to complement one's verbal communication when interacting with others. In other words, the more prepared the staff are to sign sequences of words, the better the communication support they will be able to provide to the service users.

Regarding our third research question, the training led to improvements in participants' use of KWS when supporting service users. Notably, all experimental participants signed more than the control participants, despite also being trained in KWS. There are various potential explanations for this finding. First, it is possible that experimental participants used KWS more than control participants because they had opportunities to develop fluency. Although training approaches vary, they tend to be delivered either in‐house or commercially during isolated training days. As a result, participants do not have opportunities to practice skills adequately and receive feedback, leading to a lack of application of skills to the work environment. Notably, evidence suggests that less than 20% of training is actually transferred to the job (Kazbour et al. 2013; McGee and Freds 2025). Second, based on the service's report, control participants received training at least 1 year before the study began. This could have led them to forget how to use certain signs. However, although this could be a plausible explanation, it further highlights the need for improved training procedures. In our opinion, successful training should produce a maintenance of improvements. Otherwise, it seems counterproductive to deliver training to staff members, knowing that they will not maintain the skills. Nonetheless, despite positive outcomes for the experimental participants, they did not consistently use signs throughout the training weeks, and their performance improvements were assessed only in the short term. This suggests that additional elements need to be in place in services to promote a consistent application of signing, a consideration that has been highlighted in the relevant literature. Notably, recent studies have demonstrated that some acquired signs may be lost after instruction, underlying the need for additional elements to ensure retention of KWS, as ultimately the most important test of a training programme's effectiveness lies in the successful use of KWS when supporting service users (Le Van et al. 2019; Smidt et al. 2019, 2025).

Organisational support for staff is an important factor in bringing about lasting changes in quality support to people with intellectual disabilities (Kazbour et al. 2013; Mansell et al. 2004). One way of achieving this might be via practice leadership (Bigby et al. 2020). Practice leadership involves managers having an explicit focus on the development of staff skills and, importantly, staff's everyday practice (Mansell and Beadle‐Brown 2012). Bould et al. (2018) found that a better quality of support was present in services where practice leadership was observed. Including approaches such as KWS as a focus for practice leaders might be one way to increase implementation. Specifically, a clear strategic plan for services should be in place that includes additional elements, such as ensuring that all staff members are trained in KWS, consistently modelling the use of KWS by senior staff members, incorporating elements of goal‐setting, providing feedback after naturalistic observations, employing visual reminders, and conducting refresher sessions throughout the year (Choi and Johnson 2022).

Most importantly, considering the resource implications, particularly in terms of the time required to achieve fluency, a strategic plan would be necessary to ensure that the service offers adequate time to its staff to complete the training. Based on the promising maintenance of improvements in this study, we suggest that the return on investment could be considerable if combined with additional strategies suggested above. It is also worth noting that although participants maintained their performance well above baseline, three of the four showed gradual deceleration during maintenance, with only one showing slight acceleration. This decline may be attributable to the absence of feedback during naturalistic observations. Providing brief feedback in these contexts might have helped participants sustain their performance more effectively and avoid the observed drift.

Furthermore, there was an additional noteworthy finding. Experimental participants demonstrated agility. Agility refers to our ability to ‘learn how to learn faster’ and is measured in various ways (see Meyer et al. 2021, for a detailed account). Although it has not been as widely researched as fluency, it is considered an essential outcome of PT. In this study, our participants demonstrated a gradual increase in their average responses as they transitioned from one subset to the next, even though they practised different sets of signs within each one. Moreover, their performance in the first session of each subset was higher than their performance in the first session of the previous subset, which further demonstrates agility and is known as ‘climbing bottoms’ in PT (Lindsley 2000; Meyer et al. 2021; Neely 2003). In other words, experimental participants demonstrated that they became more efficient (or agile) at learning signs as the training progressed. Overall, this is a positive finding and adds more evidence to this scarcely researched phenomenon.

Finally, it is worth noting that all participants received the same number of sessions and timings, to enable comparisons and protect the study's internal validity. However, PT encourages instructors to adjust teaching based on ongoing performance and learning data. Standard Celeration Charts are key to this process, offering precise insights. We recommend tailoring the number of timings or sessions to individual needs, as some staff may require less practice to reach fluency, while others may need more.

### 4.1 Limitations

This study had several limitations. First, one participant had to withdraw from the study as it did not align with their working hours. Considering that the focus is on making this training available to practitioners in services, it suggests to us that further refinement might be necessary to make the study more accessible to all professionals. Second, we were unable to precisely report when control participants received their training, as the service reported that the training occurred at least 1 year before the study's commencement without additional details. Third, the study took place in a single service, and we had a small sample in line with single‐case design methodologies; therefore, generalising to the broader population is not possible without additional replications. Finally, although the staff members in the service chose the signs trained, we believe that there were cases of unnecessary repetition (e.g., shower and ‘to shower’). Although a nuanced usage of all available signs would be optimal, it might have been better if other signs were chosen since this was meant to be the participants' first introduction to signs. That way, they could have potentially developed an even broader lexicon of signs, allowing them to use signs even more.

### 4.2 Future Directions

To address limitations, comparisons should be made between practitioners who have recently completed other forms of training and those receiving PT to determine whether the recency of training influences outcomes. We also suggest selecting signs that align with service users' needs while minimising overlap. Furthermore, an evaluation of whether the training could be delivered in a shorter timeframe while maintaining its effectiveness is warranted. Finally, a longer assessment of KWS retention (e.g., 12+ weeks) would be optimal and aligned with the existing literature on KWS training (Smidt et al. 2019, 2025).

## Funding

This research was funded by the British Academy (grant number SRG23\230886).

## Ethics Statement

The study received ethical approval from the University of Kent ethics committee.

## Conflicts of Interest

The second author was a staff member at the service where the research occurred. However, steps were taken to mitigate biases, ensuring data integrity and objective analysis. The other authors declare no conflicts of interest.

## Data Availability

The data that support the findings of this study are openly available in Figshare at https://figshare.com/. For the purpose of open access, the author(s) have applied a Creative Commons Attribution (CC BY) licence to any Author Accepted Manuscript version arising. Additional resources, including the SCCs, fidelity checklists, participants' datasheets, and survey, are available via this link https://doi.org/10.6084/m9.figshare.c.8245924.

## Endnotes

^1^
Free is another word for think (which is free from one's observation).
